# Malaria vaccine candidate based on Duffy-binding protein elicits strain transcending functional antibodies in a Phase I trial

**DOI:** 10.1038/s41541-018-0083-3

**Published:** 2018-09-28

**Authors:** Kavita Singh, Paushali Mukherjee, Ahmad Rushdi Shakri, Ankita Singh, Gaurav Pandey, Meenakshi Bakshi, Geetanjali Uppal, Rajender Jena, Ankita Rawat, Purnima Kumar, Rukmini Bhardwaj, Syed Shams Yazdani, Dhiraj Hans, Shantanu Mehta, Ajay Srinivasan, K. Anil, R. L. Madhusudhan, Jaya Patel, Amit Singh, Rajeshwar Rao, Santosh Gangireddy, Rudrappa Patil, Swarnendu Kaviraj, Sanjay Singh, Darrick Carter, Steve Reed, David C. Kaslow, Ashley Birkett, Virander S. Chauhan, Chetan E. Chitnis

**Affiliations:** 1 Multi-Vaccines Development Program, New Delhi, India; 2 International Centre for Genetic Engineering and Biotechnology, New Delhi, India; 3 USBT, Guru Gobind Singh Indraprastha University, New Delhi, India; 4 Syngene International Ltd, Bangalore, India; 5 Gennova Biopharmaceuticals Ltd, Pune, India; 6 Infectious Disease Research Institute, Seattle, WA USA; 7 PATH Malaria Vaccine Initiative, Seattle, WA USA; 8 Institut Pasteur, Paris, France

## Abstract

Reticulocyte invasion by *Plasmodium vivax* requires interaction of the Duffy-binding protein (PvDBP) with host Duffy antigen receptor for chemokines (DARCs). The binding domain of PvDBP maps to a cysteine-rich region referred to as region II (PvDBPII). Blocking this interaction offers a potential path to prevent *P. vivax* blood-stage growth and *P. vivax* malaria. This forms the rationale for development of a vaccine based on PvDBPII. Here we report results of a Phase I randomized trial to evaluate the safety and immunogenicity of recombinant PvDBPII formulated with glucopyranosyl lipid adjuvant-stable emulsion (GLA-SE). Thirty-six malaria-naive, healthy Indian male subjects aged 18–45 years were assigned into three cohorts corresponding to doses of 10, 25 and 50 µg of PvDBPII formulated with 5 µg of GLA-SE. Each cohort included nine PvDBPII/GLA-SE vaccinees and three hepatitis B control vaccine recipients. Each subject received the assigned vaccine intramuscularly on days 0, 28 and 56, and was followed up till day 180. No serious AE was reported and PvDBPII/GLA-SE was well-tolerated and safe. Analysis by ELISA showed that all three doses of PvDBPII elicited antigen-specific binding-inhibitory antibodies. The 50 µg dose elicited antibodies against PvDBPII that had the highest binding-inhibitory titres and were most persistent. Importantly, the antibody responses were strain transcending and blocked receptor binding of diverse PvDBP alleles. These results support further clinical development of PvDBPII/GLA-SE to evaluate efficacy against sporozoite or blood-stage challenge in controlled human malaria infection (CHMI) models and against natural *P. vivax* challenge in malaria endemic areas.

## Introduction

Among the five species of *Plasmodium* that infect humans, *P. falciparum* and *P. vivax* are the most prevalent. *P. vivax* is the predominant species responsible for majority of malaria cases outside sub-Saharan Africa.^1^ Most cases of vivax malaria occur in the WHO South-East Asia Region (58%), followed by the WHO Eastern Mediterranean Region (21%) and the WHO African Region (10%). In 2016, 85% of estimated vivax malaria cases occurred in just five countries (Afghanistan, Ethiopia, India, Indonesia and Pakistan).^2^ *P. vivax* is responsible for significant morbidity with a recent estimate suggesting that 13.8 million (95% confidence interval: 10.3–18.4 million) vivax cases occurred in 2014.^3^ Importantly, recent studies have reported that *P. vivax* can also cause severe disease and mortality.^4^ Thus, there has been renewed interest in strategies to protect against and eventually eliminate *P. vivax* globally.

Tools used to control *P. falciparum* are less effective against *P. vivax* due to its unique biology. For example, *P. vivax* forms latent hypnozoite stages in the liver that can cause blood-stage infections weeks or months after the initial infection. *P. vivax* hypnozoites contribute significantly to the prevalence of *P. vivax* malaria. They cannot be detected and are therefore difficult to eliminate. Moreover, *P. vivax* gametocytes appear early during blood-stage infection so that transmission takes place even before clinical symptoms develop and the patient can be treated to clear the infection. An effective vaccine that can protect against *P. vivax* malaria and reduce transmission would be a valuable additional tool for control efforts that can help accelerate progress towards elimination of *P. vivax*.

The blood-stage of the malaria parasite life cycle, which involves repeated cycles of red cell invasion by merozoites, intracellular replication and egress, is responsible for all the clinical symptoms of malaria. The invasion of human red blood cells by *P. vivax* is mediated by interaction of the *P. vivax* Duffy-binding protein (PvDBP) with its receptor, the Duffy antigen receptor for chemokines (DARCs).^5–9^ A conserved, cysteine-rich region of PvDBP referred to as region II (PvDBPII) serves as the functional receptor-binding domain that binds DARC to mediate invasion by *P. vivax* merozoites.^9^ Duffy-negative individuals largely remain resistant to *P. vivax* infection.^5^ Although PvDBPII is polymorphic, the binding residues that interact with DARC appear to be highly conserved across different *P. vivax* strains.^10^ This observation suggests that PvDBPII contains potential cross-reactive epitopes to which strain-transcending inhibitory antibodies might be elicited. Indeed, individuals residing in endemic areas have been shown to develop high-titre binding-inhibitory antibodies against PvDBPII that block DARC binding by diverse PvDBPII alleles with similar efficiency.^11^ Importantly, the acquisition of such binding-inhibitory antibodies is associated with protection against *P. vivax* infection.^11^ These observations provide the immuno-biological and mechanistic rationale for the development of a subunit vaccine based on PvDBPII to protect against *P. vivax* malaria.

Methods to express recombinant PvDBPII in *Escherichia coli* followed by isolation from inclusion bodies under denaturing conditions, refolding and purification by ion exchange chromatography have been developed and were described earlier.^12^ Preclinical studies with PvDBPII formulated with glucopyranosyl lipid adjuvant-stable emulsion (GLA-SE), a synthetic Toll-like receptor 4 agonist (a proprietary adjuvant from Infectious Disease Research Institute, Seattle, USA), have shown that the vaccine candidate elicits high-titre anti-PvDBPII antibodies that block receptor binding of diverse PvDBPII alleles with high efficiency.^13^ Here we report the results of a Phase I, first-in-human trial that was conducted to evaluate the safety and immunogenicity of escalating doses of recombinant PvDBPII formulated with GLA-SE (PvDBPII/GLA-SE) in malaria-naive adults. We report that the PvDBPII/GLA-SE vaccine candidate is safe and immunogenic. Importantly, immunization with PvDBPII/GLA-SE elicited high-titre anti-PvDBPII antibodies with functional biological activity. PvDBPII/GLA-SE-induced antibodies were able to inhibit binding of both homologous and heterologous variants of recombinant PvDBPII to DARC in an in vitro receptor-binding assay. These observations support further clinical development of a PvDBPII-based vaccine for *P. vivax* malaria.

## Results

### Study participants enrolled in phase 1 trial to evaluate PvDBPII/GLA-SE in a single-blind, dose-escalation study

**Fig. 1:**
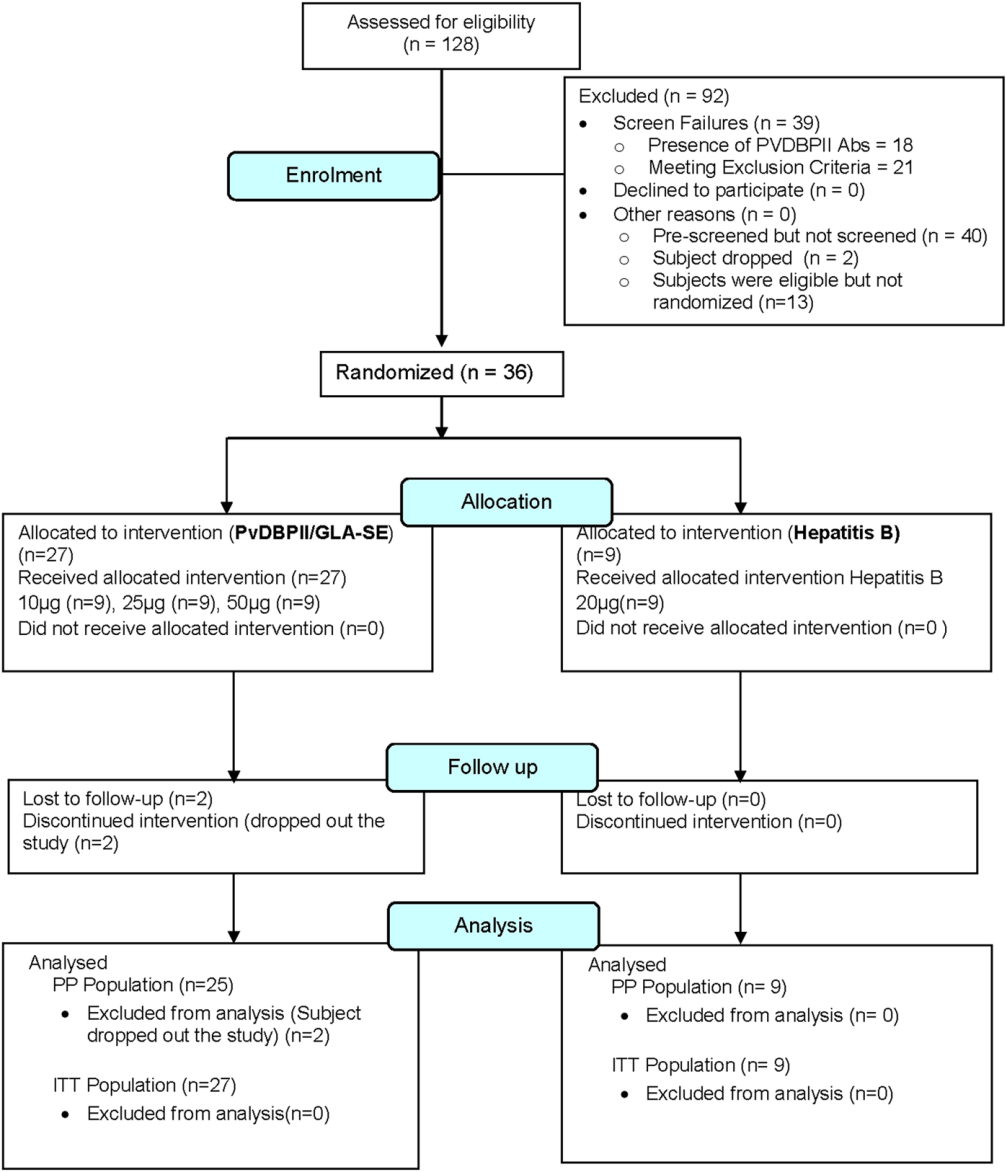
Flow chart of study design and volunteer recruitment

A total of 128 male subjects were evaluated for the presence of detectable PvDBPII antibodies at the first screening visit. From the 110 PvDBPII antibody negative subjects, 71 were further screened at the second screening visit for haematological and biochemical laboratory parameters, and predefined inclusion and exclusion criteria, as per approved protocol. From 49 subjects who met the enrolment criteria, 36 subjects were randomized in a 3:1 scheme in 3 cohorts. Each cohort had nine subjects assigned to a test arm and three subjects to a control arm. Subjects in the test arms received intramuscular doses of 10, 25 or 50 µg of PvDBPII with a constant dose of GLA-SE (5 µg of GLA), whereas subjects in the control arms received GeneVac-B (hepatitis B vaccine from Serum Institute of India). The baseline demographic characteristics of the three PvDBPII dose groups and GeneVac-B control vaccine group were comparable (Table 1). There was no significant difference between the test and control groups with respect to weight, height and age across all doses. The first subject was enrolled in October 2016 and the last subject completed the protocol-specified last visit, i.e., day 180 after first vaccination in July 2017. The study was stopped after the last subject successfully completed the last follow-up visit. Subjects in higher PvDBPII dose cohorts were immunized with an escalated vaccine dose only after review and receipt of written recommendation from an independent Data Safety Monitoring Board (DSMB), overseeing the trial as per the predefined DSMB charter. One subject each in cohorts 1 and 2, respectively, dropped out of the study before the second immunization visit. Both subjects discontinued due to personal reasons unrelated to any adverse event (AE). All remaining 34 subjects received all 3 immunizations. Complete subject disposition is provided in Fig. 1. The intention-to-treat (ITT) population includes all 36 subjects (received at least 1 dose of either of the study vaccines), whereas the per protocol (PP) population (subset of the ITT, who completed the study without any major protocol violations) includes 34 subjects.

**Table 1:** Demographics and baseline characteristics in 10, 25 and 50 µg PvDBPII vaccine and 20 µg Hepatitis B vaccine groups–ITT Population

Parameter	Statistics/category	PvDBPII (10 µg) *N* = 9	PvDBPII (25 µg) *N* = 9	PvDBPII (50 µg) *N* = 9	Hepatitis B (20 µg) *N* = 9
Age (in years)	*N*	9	9	9	9
	Mean ± SEM	29.78 ± 2.09	34.56 ± 1.85	27.56 ± 1.74	34.67 ± 2.12
	Median	28	36	30	35
	(Min, Max)	(22.00, 42.00)	(25.00, 41.00)	(18.00, 33.00)	(26.00, 45.00)
	Range	20	16	15	19
	*p-Value	0.051			
Weight (kg)	*N*	9	9	9	9
	Mean ± SEM	62.87 ± 2.77	67.84 ± 2.26	66.94 ± 2.36	68.61 ± 2.03
	Median	63	70	66.2	66.1
	(Min, Max)	(51.00, 74.00)	(56.00, 76.00)	(57.30, 77.00)	(61.00, 79.30)
	Range	23	20	19.7	18.3
	*p-Value	0.442			
Height (cm)	*N*	9	9	9	9
	Mean ± SEM	166.67 ± 1.76	170.11 ± 1.97	169.67 ± 1.77	168.22 ± 2.67
	Median	167	171	169	166
	(Min, Max)	(158.00, 175.00)	(161.00, 179.00)	(159.00, 176.00)	(159.00, 183.00)
	Range	17	18	17	24
	*p-Value	0.566			

*Max* maximum, *Min* minimum

*N* = total number of subjects randomized

*p-Value: between study group comparison is performed using Kruskal–Wallis test

### PvDBPII/GLA-SE was safe in malaria-naive Indian male adult volunteers

All immunizations were generally well tolerated and no serious AE (SAE) or death was reported during the study. There was no immediate local reactogenicity observed post immunization in any of the study groups. In this study, a total 68 AEs were recorded including 67 unsolicited AEs. Of these, 14 AEs were observed in the PvDBPII 10 µg group, 15 AEs in the PvDBPII 25 µg group, 19 AEs in the PvDBPII 50 µg group and 20 AEs in the GeneVac-B control vaccine group. The incidence of AEs in different doses of the test vaccine groups varied from 20% to 28% as compared with 29% observed in the control group (Table 2). There was no subject withdrawal due to an AE. Only one local solicited AE (mild pain at injection site) was reported (Supplementary Table S1). In the PvDBPII 10 µg dose group after the first dose, one subject experienced pain at the injection site, which was assessed to be temporary and mild in severity. No intervention was needed as the AE resolved without sequelae and subsequent doses of the study vaccine were administered as scheduled. No solicited systemic AEs were reported in the study (Supplementary Table S1).

**Table 2:** Number of subjects reported with AEs by study group

Description	PvDBPII (10 µg)	PvDBPII (25 µg)	PvDBPII (50 µg)	Hepatitis B (20 µg)
AEs (*N*)	14	15	19	20
Solicited AEs	1 (7.14)	0	0	0
Unsolicited AEs	13 (92.86)	15 (100)	19 (100)	20 (100)
- Lab abnormalities	12 (92.31)	15 (100)	19 (100)	20 (100)
- Clinical AE (rash)	1 (7.69)	0	0	0
SAEs (*n*)	0 (0.00)	0 (0.00)	0 (0.00)	0 (0.00)
AE lost to follow-up (*n*)*	0 (0.00)	4 (26.67)	0 (0.00)	0 (0.00)

*AE* adverse event, *N*, total number of AEs, *n*, number of events for each study group, *SAE* serious AE; % = (*n*/*N*)100

*All four AEs lost to follow-up were reported in subject R202

Most of the unsolicited AEs were common or similar across all study groups. A total of 31 subjects (86.11%) experienced 67 unsolicited AEs during the study. Of these, 66 clinical laboratory-related AEs were found to be unrelated to study or control vaccines (PvDBPII or GeneVac-B vaccine) and only 1 (01) clinical AE (rash) was judged to be related to PvDBPII (10 µg) (Supplementary Table S2). The rash developed on the medial aspect of the right forearm 2 h after administration of the test vaccine to the deltoid region of the left forearm. Based on the temporal association of rash with vaccine administration (appearance of rash within 2 h of immunization) in terms of time of occurrence, resolution and with no prior subject history of allergies, the AE was adjudged as vaccine related. The event was classified as grade 1 (mild) in severity and resolved within an hour without any sequelae. The subject received two subsequent vaccine doses as scheduled at visit 6 and visit 11 with no repeat incidence of rash. Sixty-three of the 67 unsolicited AEs resolved without intervention or sequelae. The outcome of remaining four AEs were unknown as the subjects were lost to follow-up.

Most of the AEs reported in this study were judged to be mild or moderate in severity (45 AEs were mild; 19 AEs were moderate). Only three laboratory-related AEs were grade 3 or above in severity. These included two incidences of Grade 3 increase in serum sodium (hypernatremia) and one incidence of Grade 4 increase in serum potassium levels (hyperkalemia). Grade 3 hypernatremia was reported at Day 180 in one subject each in test and in control groups, respectively. Clinical assessment of these subjects showed no associated clinical signs or symptoms. Repeat investigations revealed normal serum sodium levels. No medical interventions were required and the AEs were considered resolved without sequelae. Thus, both events were considered clinically not significant and not related to study vaccine administration. An isolated incidence of Grade 4 hyperkalemia not related to study vaccine was reported in the 50 µg PvDBPII dose group, 7 days post first dose. This event was considered clinically not significant, as there was no correlation with any clinical signs or symptoms, required no medical intervention, resolved spontaneously (normal serum potassium levels seen on repeat testing after 4 days) without sequelae and with no association with subsequent doses. As a safety measure, serum potassium levels were monitored before subsequent doses and were found to be within normal range both pre- as well as after subsequent doses. Thus, the event was considered as an isolated incident not related to the study vaccine.

Other important laboratory abnormalities included increased liver enzyme levels (alanine transferase and aspartate transferase) and blood glucose levels, and decreased haemoglobin levels observed in some subjects in both test and control groups (Supplementary Table S2). These AEs were mild or moderate in severity, not associated with any clinical signs or symptoms, and showed no association with repeat dosing or increasing dose of the test vaccine and were hence considered as clinically not significant and not related to study vaccines. All the laboratory abnormalities resolved spontaneously or showed a resolving trend by end-of-the study.

### PvDBPII/GLA-SE induced seroconversion in study participants

The humoral immune response against PvDBPII was evaluated for the PP Population. Baseline anti-PvDBPII titres did not differ significantly between the three PvDBPII dose groups. Seroconversion was defined as ≥ 4-fold increase in antibody titre compared with baseline antibody titre. Seroconversion rates after two doses at Day 56 and after three doses at Day 84 were 100%, as all subjects who received different doses of PvDBPII showed ≥ 4-fold increase in titres post immunization. None of the GeneVac-B control vaccine recipients seroconverted to recognize PvDBPII.

### Antibody titres induced by PvDBPII/GLA-SE formulations in study participants

**Fig. 2:**
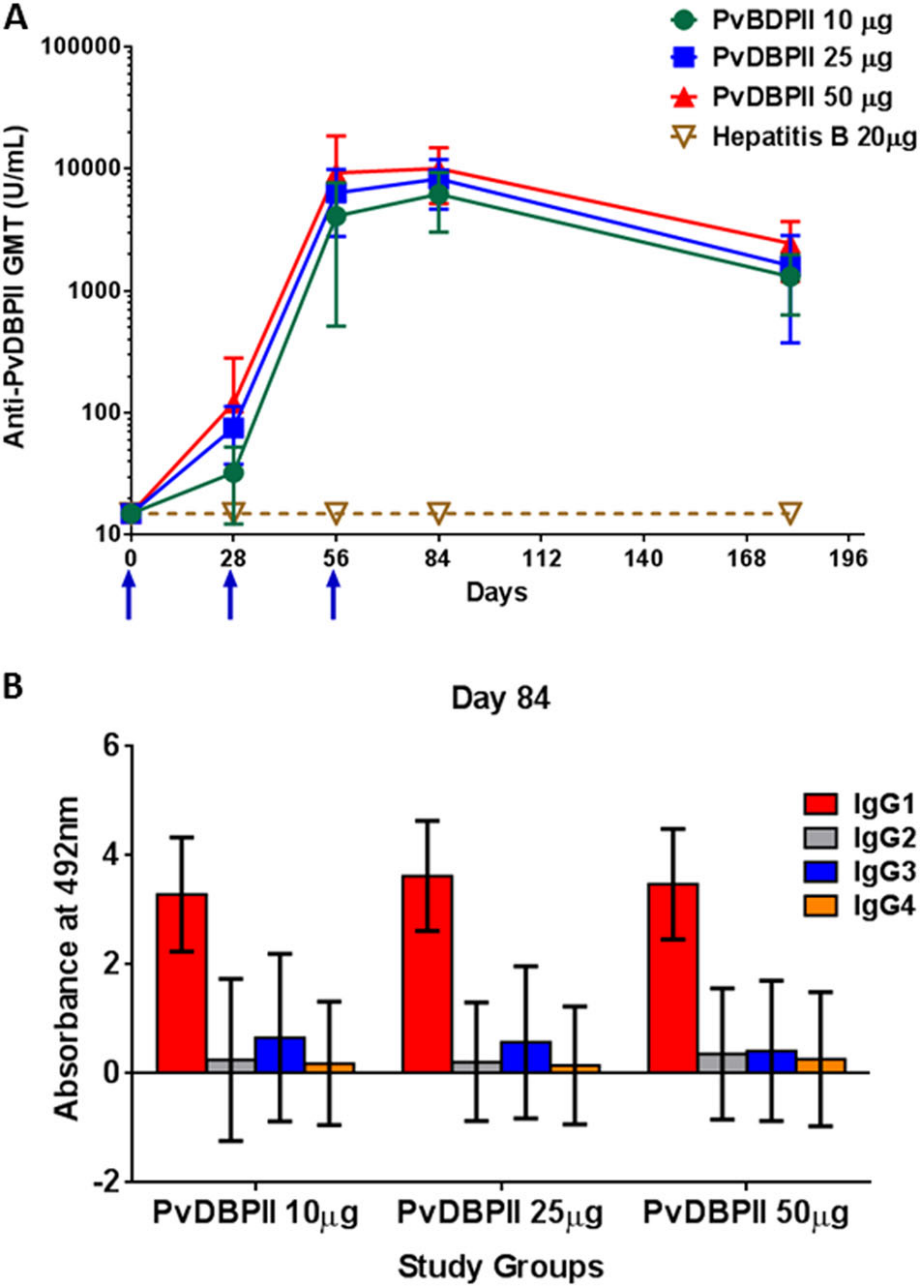
Humoral response to immunization with PvDBPII formulated with GLA-SE. **a** Kinetics of PvDBPII-specific Geometric Mean antibody titres for all Study Groups (PP Population). Geometric mean antibody levels (U/mL) to PvDBPII measured by ELISA in sera collected from groups immunized with 10, 25, or 50 μg PvDBPII/GLA-SE and Hepatitis B vaccine. Study participants were immunized on Days 0, 28 and 56, and sera were collected on Days 0, 28, 56, 84 and 180. Antibody levels measured by ELISA were expressed as Geometric Mean Titres (GMT) with 95% confidence interval for the nine subjects. **b** Serum IgG subclass responses to PvDBPII in three PvDBPII dose groups (PP Population). Geometric means with SD are shown

The kinetics of the anti-PvDBPII IgG antibody response were followed for 6 months in all three dose groups of PvDBPII. Negligible levels of PvDBPII-specific antibodies were observed before immunization, with baseline geometric mean titre (GMT) of ≤ 15. All subjects in the GeneVac-B control vaccine group maintained baseline level of antibody at all time points. The kinetics of the antibody responses were similar for all three dose groups (Fig. 2a). A modest increase in antibody titres occurred after the first dose in all PvDBPII dose groups. However, after the second dose an increase in anti-PvDBPII GMT) occurred at Day 56 in all three PvDBPII dose groups with no statistically significant differences between the groups (Fig. 2). PvDBPII GMT was significantly higher in all three PvDBPII dose groups compared with GeneVac-B control vaccine group (for all comparisons, *p* = 0.0002).

Following the third dose given on Day 56, PvDBPII antibody responses increased in all PvDBPII dose groups as determined at Day 84. The 50 µg PvDBPII dose group continued to exhibit higher GMT titres (8889.0; 95% Cl: 5814.0–13595.5) compared with the 25 µg (7604.7; 95% Cl: 5266.3–10981.6) and the 10 µg (5539.5; 95% Cl: 3638.9–8432.8) PvDBPII dose groups. No statistically significant differences were observed in enzyme-linked immunosorbent assay (ELISA) titres of Day 84 sera between the three PvDBPII dose groups following the third dose on Day 56. GMT responses of the three PvDBPII dose groups observed on Day 84 were significantly greater than the GeneVac-B control vaccine group (*p* = 0.0002). After three doses, the predominant response to PvDBPII was IgG1 subclass followed by IgG3 and IgG2, and low levels of IgG4 (Fig. 2b).

During follow-up, after the third dose, anti-PvDBPII GMT titres declined by Day 180 (Fig. 2a) but were significantly higher for all three PvDBPII dose groups compared with Day 0 titres. The distribution of PvDBPII antibody titres at Day 84 and Day 180 were further analysed using reverse cumulative distribution curves (Supplementary Figure S1). Compared with 10 µg and 25 µg, the 50 µg PvDBPII dose induced a stronger immune response as evidenced by a shift of the distribution curve to the right following three doses, as measured at Day 84 and at Day 180 (Supplementary Figure S1). The effect of PvDBPII dose on antibody responses at the population level can be seen in the reverse cumulative distribution curves.

### PvDBPII-specific antibodies recognize native antigen in late-stage *P. vivax* schizonts

**Fig. 3:**
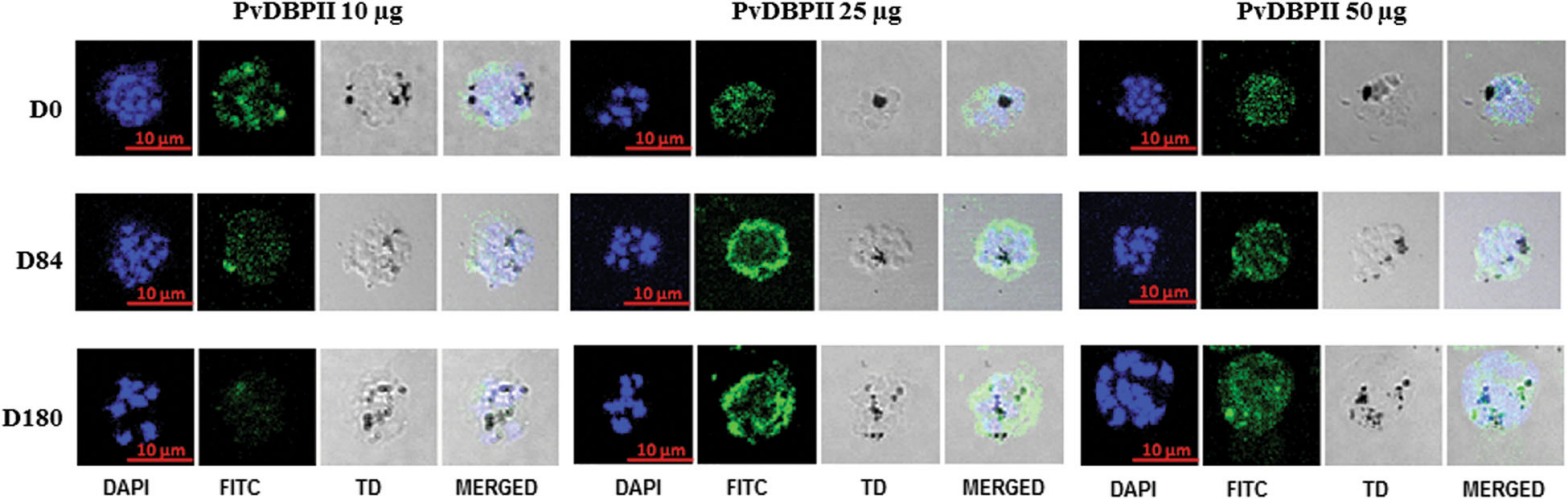
Reactivity of sera from PvDBPII immunized subjects with *P. vivax*-infected erythrocytes obtained from vivax malaria patients. Representative images are shown from each of the three PvDBPII dose groups (PP Population)

To determine whether PvDBPII-specific antibodies are capable of recognizing native antigen in *P. vivax* parasites, Day 0, 84 and 180 sera were tested by immunofluorescence assays (IFA) using *P. vivax* parasite slides prepared from blood of *P. vivax*-infected patients. PvDBPII-specific antibodies in Day 84 and Day 180 serum samples reacted with mature schizonts, showing punctate and peripheral staining (Fig. 3). In the 10 µg PvDBPII dose group, seven out of eight Day 84 and Day 180 serum samples reacted strongly with *P. vivax* schizonts (Fig. 3). In the 25 µg PvDBPII dose group, eight out of eight sera from Day 84 and Day 180 showed strong reactivity with *vivax* malaria parasites. In the 50 µg PvDBPII dose group, eight out of nine sera from Day 84 sera and Day 180 reacted strongly with the parasites. Some of the Day 0 sera showed low level of diffused nonspecific reactivity to the parasite (10 µg: 5/8 sera; 25 µg: 3/8 sera and 50 µg: 5/8 sera) as shown in the representative images in Fig. 3. Although the qualitative IFA results support the ELISA data and confirm seroconversion to PvDBPII vaccine, the observed low level nonspecific reactivity of some Day 0 sera with parasites makes it difficult to draw any specific conclusion about end point titres for IFA reactivity.

### PvDBPII-specific antibodies block binding of PvDBPII with DARC

Next, to determine whether anti-PvDBPII antibodies in serum samples were able to block interaction of PvDBPII with its receptor DARC, Day 0, 84 and 180 serum samples were tested in an ELISA-based binding-inhibition assay (BIA). Sera were tested at different dilutions from 1:10 to 1:10,000, with percentage binding inhibition calculated for each dilution. Fifty percent binding-inhibition titres were determined from percent inhibition vs dilution curves. Day 0 samples did not demonstrate any binding-inhibitory activity. Day 84 sera samples from the 10, 25 and 50 µg dose groups showed binding-inhibitory activity > 60% at a dilution of 1:10 except for one serum sample in the 25 µg dose group that showed weak (30%) binding inhibition (Supplementary Figure S2).

**Fig. 4:**
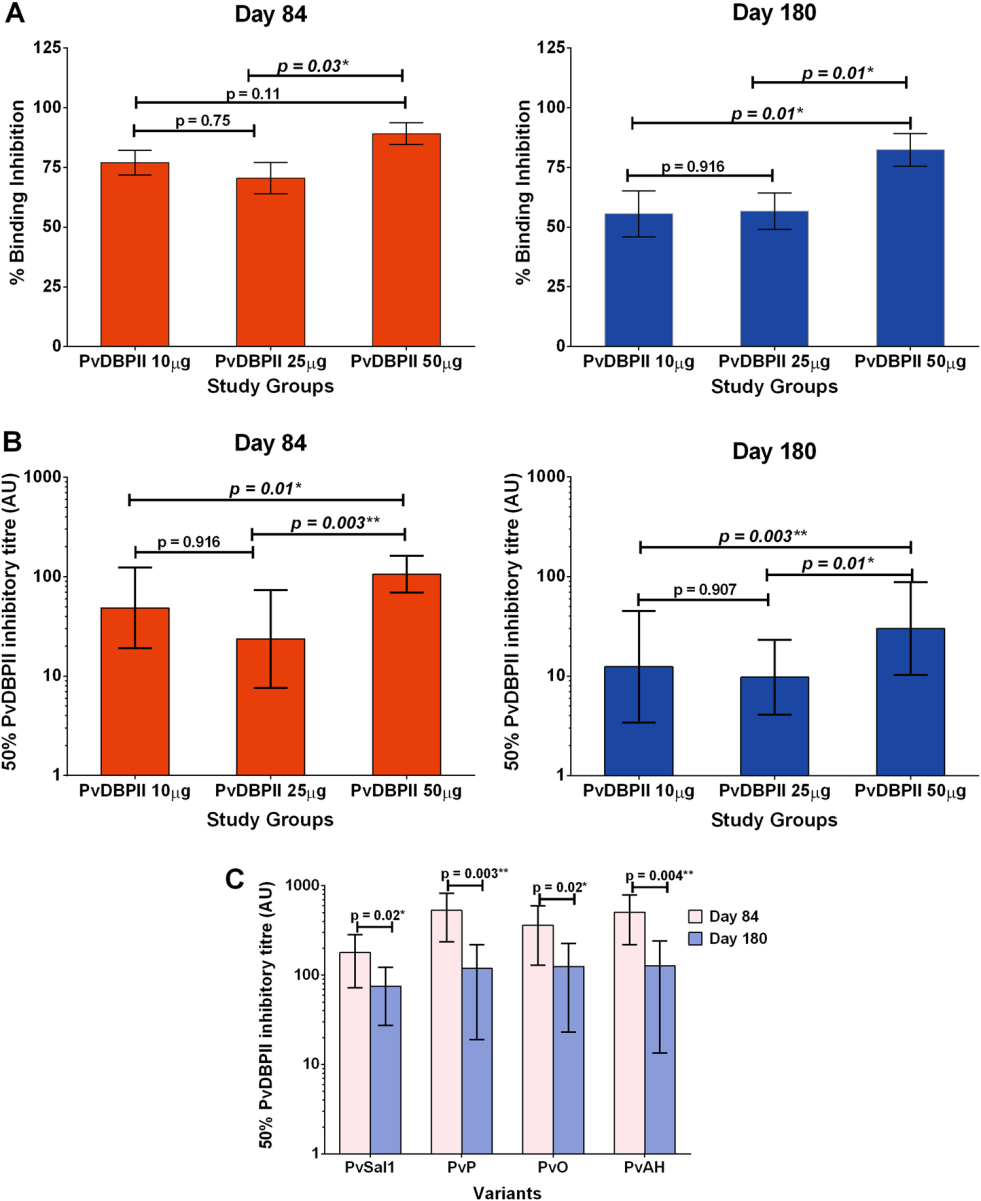
Inhibition of binding of recombinant PvDBPII variants to the Duffy antigen receptor for chemokines (DARC) using an ELISA-based assay. **a** Mean percentage binding inhibition at Day 84 and Day 180 for sera from groups immunized with PvDBPII (Sal I) at 10, 25 and 50 µg, and tested at a 1:10 sera dilution (PP Population). **b** 50% Binding-inhibitory titres for inhibition of PvDBPII-DARC binding using Day 84 and Day 180 sera from groups immunized with 10, 25 and 50 μg of PvDBPII formulated with GLA-SE. **c** Inhibition of DARC binding by PvDBPII variants using sera from groups immunized with 10, 25 and 50 μg of PvDBPII formulated with GLA-SE. Means with standard deviations are shown

The mean binding-inhibitory activity on Day 84 at the 1:10 dilution was notably higher in the 50 µg PvDBPII dose group (89.20 ± 4.49%) compared with the 10 µg PvDBPII dose (77.03 ± 5.11%; *p* = 0.01) and the 25 µg PvDBPII dose group (70.49 ± 6.54%; *p* = 0.03) (Fig. 4a). The GeneVac-B control vaccine group showed no binding-inhibitory activity.

Mean binding-inhibitory activity of Day 180 sera was markedly lower in the 10 µg PvDBPII dose group (55.59 ± 9.57%) and the 25 µg PvDBPII dose group (56.65 ± 7.65%) compared with the 50 µg PvDBPII dose (82.33 ± 6.89%) tested at the 1:10 dilution. The level of inhibition of Day 180 sera in the 50 µg PvDBPII dose group (82.33 ± 6.89%) was marginally lower than Day 84 sera (89.20 ± 4.49%) (Fig. 4b).

All Day 0 serum samples showed baseline binding-inhibitory titre values. A dose response was observed in the 50% PvDBPII binding-inhibitory GMTs at Day 84 in PvDBPII-vaccinated subjects. The 50 µg PvDBPII dose showed significantly higher 50% binding-inhibition titres (GMT: 105.9; 95% Cl, 69.2, 162.0) compared with the 10 µg PvDBPII dose (GMT: 48.6; 95% Cl, 19.0, 124.1) and the 25 µg dose group (GMT: 23.7; 95% Cl, 7.6, 73.6) (Fig. 4). At Day 180, the 50% binding-inhibition GMT declined in all PvDBPII dose groups. The 50 µg PvDBPII dose group showed the least decline in binding-inhibitory titres from Day 84 to Day 180 (Fig. 4). These results suggest that PvDBPII is capable of eliciting high-titre binding-inhibitory antibodies.

Finally, we tested the Day 84 anti-PvDBPII sera raised against PvDBPII Sal I allele (vaccine candidate) for inhibition of binding of PvDBPII variants (PvAH, PvO and PvP) derived from *P. vivax* isolates from Papua New Guinea.^14^ Day 84 sera inhibited binding of all variants tested (Fig. 4) with comparable 50% binding-inhibition titres (Supplementary Figure S3). At Day 180, a significant drop in 50% binding-inhibition titres was observed compared with the Day 84 samples for all variants (Fig. 4).

### Correlation seen between BIA and ELISA antibody response

There was no significant positive correlation between anti-PvDBPII ELISA titres and percentage binding inhibition (at 1:10 sera dilution) at Day 84 in the 10 μg PvDBPII dose group (*r* = 0.24, *p* = 0.958), 25 μg (*r* = 0.38, *p* = 0.369) or 50 μg PvDBPII dose groups (*r* = 0.52, *p* = 0.161). However, at Day 180, the correlation between binding inhibition at 1:10 and ELISA titre was found to be statistically significant for the 50 μg PvDBPII dose group (*r* = 0.82, *p* = 0.004) (Supplementary Figure S4), but not for the 10 µg and 25 µg PvDBPII dose groups. In addition, correlation was observed between PvDBPII ELISA titres and 50% PvDBPII binding-inhibitory titres at Day 180 for 10 μg (*r* = 0.81, *p* = 0.01) and 50 μg PvDBPII dose groups (*r* = 0.83, *p* = 0.007) (Supplementary Figure S4). No correlation was seen between PvDBPII ELISA titres and binding-inhibitory titres in 25 μg PvDBPII dose groups.

## Discussion

This study was designed to evaluate the safety and immunogenicity of three increasing doses of a recombinant *P. vivax* malaria vaccine candidate, PvDBPII, formulated with the adjuvant GLA-SE. The vaccine candidate, PvDBPII/GLA-SE, which was tested in healthy Indian male adults, was safe and well tolerated. No significant immediate reactogenicity was observed within the first hour post immunization at all three dose levels (10, 25 and 50 μg PvDBPII formulated with GLA-SE [5 μg of GLA]). No SAE was observed and no subject withdrew or was withdrawn from the study on account of safety. A single mild local solicited AE (pain at injection site) after the first dose of 10 µg PvDBPII was reported. No systemic solicited AEs were observed. There were similar incidences and intensity of unsolicited AEs among the subjects who received PvDBPII/GLA-SE and those who received GeneVac-B (control hepatitis B vaccine). No clinically significant difference was observed in haematology, biochemistry and urinalysis parameters post vaccination. Vital signs showed no significant changes throughout the study. No abnormal findings were observed during the post-study physical examination. There was no difference in the number of subjects who had biological values outside the normal ranges for different haematological and biochemical parameters in the PvDBPII/GLA-SE and control vaccine group. It may be noted that abnormal laboratory values were defined according to international normal ranges, which makes the safety assessments less tailored to the population of interest.

Immunogenicity data demonstrate that all three doses of PvDBPII (10, 25 and 50 µg) elicited antigen-specific and receptor-blocking serum antibody responses. The 50 µg dose elicited the most persistent binding-inhibitory antibodies against PvDBPII. Administration of PvDBPII formulated with GLA-SE elicited significant PvDBPII-specific humoral responses at all time points in all dose groups. A dose-dependent increase in antibody responses was observed, although this was not found to be statistically significant. The 50 µg PvDBPII/GLA-SE vaccine dose elicited the highest antibody response against PvDBPII. The antibody response increased after the second dose but there was no further increase in antibody titres after the third dose. It may be useful to increase the spacing between second and third doses to allow boosting with the third dose. Between-group comparisons did not show statistically significant differences in GMTs at any post-dose time points. All subjects in each of the three PvDBPII dose groups exhibited ≥ 4-fold increases in anti-PvDBPII antibody titres. Antibodies induced by the PvDBPII/GLA-SE vaccine recognized the native PvDBPII protein in *P. vivax* parasite by IFA. However, as low-level nonspecific reactivity of some Day 0 serum samples was observed with the parasite, it was not possible to determine IFA titres and no quantitative conclusions were made from the IFA data. In future studies, reactivity of volunteer sera with *P. vivax* blood stages by IFA should be included as an exclusion criterion in addition to reactivity with PvDBPII by ELISA assay.

Antibodies collected on Days 84 and 180 following three doses of PvDBPII were found capable of inhibiting binding of PvDBPII to DARC in an in vitro binding assay. Highest binding-inhibitory activity was observed in the 50 µg PvDBPII dose group. Although the immunogenicity data show that all three doses of PvDBPII (10, 25 and 50 µg) elicited antigen-specific and receptor-blocking serum antibody responses, the 50 µg dose elicited the highest binding-inhibition titres and most persistent binding-inhibitory antibodies against PvDBPII as measured with Day 180 sera. Based on the binding-inhibition data of sera from 50 µg PvDBPII dose group, this dose is recommended for further studies. Importantly, both Day 84 and Day 180 sera inhibited binding not only of the homologous PvDBPII Sal I allele but also inhibited binding of three other PvDBPII alleles (P, O and AH), which are commonly found in Papua New Guinea^10,14^ with similar 50% binding-inhibition titres. Structure–function studies have previously indicated that the DARC-binding site within PvDBPII is conserved and predicted that it should be possible to elicit strain-transcending binding-inhibitory antibodies against PvDBPII. The observations reported here demonstrate that the *P. vivax* vaccine candidate, PvDBPII/GLA-SE can elicit strain-transcending binding-inhibitory antibodies and should be effective against diverse *P. vivax* strains. Whether the levels of antibodies elicited against PvDBPII are sufficient to control blood-stage parasite growth in vivo and provide protection remains to be tested. A previous Phase I trial that used viral vectors including modified vaccinia Ankara followed by the simian adenovirus, ChAd63, in a prime-boost immunization strategy to deliver PvDBPII also raised high-titre, strain-transcending binding-inhibitory antibodies that blocked receptor binding of diverse PvDBPII domains.^15^ The binding-inhibitory titres obtained using viral vectors were comparable to those reported here. The Phase I trial described here is the first report of a human clinical trial using a recombinant protein-based vaccine targeting PvDBPII.

Ideally, a *P. vivax* blood-stage vaccine should elicit antibodies that inhibit blood-stage growth with sufficiently high efficiency to clear blood-stage parasites rapidly and protect against clinical symptoms. Moreover, an ideal blood-stage vaccine would block blood-stage growth so efficiently that it would limit development of gametocytes and have an impact on transmission. No correlates of protection have been defined for *P. vivax* so far and we do not know the titres of antibodies against PvDBPII that are required to inhibit *P. vivax* invasion into reticulocytes efficiently to block blood-stage growth. It is thus difficult to judge whether the antibody titres achieved here will be effective in protecting against *P. vivax* in the field. The next steps are to test the efficacy of immunization with PvDBPII against controlled challenge with *P. vivax* blood stages and against natural challenge in the field. As we make progress in clinical development of PvDBPII and test efficacy in *P. vivax* blood-stage challenge models, as well as natural challenge in field trials, it will become possible to define anti-PvDBPII binding-inhibition titres needed to provide protection against *P. vivax* malaria. Definition of such neutralization titres will greatly aid the development of PvDBPII-based malaria vaccines.

In recent years *P. vivax* blood-stage infections have been reported in Duffy-negative individuals from multiple locations in Africa.^16–18^ The invasion pathways used by *P. vivax* strains for invasion of Duffy-negative reticulocytes are not clearly understood. It is possible that low-level transient expression of Duffy antigen in early-stage reticulocytes in some Duffy-negative individuals might enable *P. vivax* invasion through the Duffy pathway.^19^ In such cases, a vaccine based on PvDBPII could protect Duffy-negative individuals against *P. vivax* malaria. However, if *P. vivax* uses an alternative invasion pathway that is mediated by parasite proteins such as the PvDBP homologue, PvEBP,^20^ or by members of the reticulocyte-binding protein family (PvRBPs),^21,22^ it may be necessary to include other antigens to a *P. vivax* blood-stage vaccine to inhibit invasion by these alternative invasion pathways. Further investigations into molecular interactions used for reticulocyte invasion by *P. vivax* are needed to distinguish between these possibilities.

In conclusion, results of the Phase I trial indicate that all three dose levels of the PvDBPII/GLA-SE vaccine candidate are safe and immunogenic in malaria-naive adult males. In addition, functional analyses of the antibodies elicited by PvDBPII/GLA-SE demonstrate that they block binding of PvDBPII to its receptor DARC in an in vitro binding assay. These observations provide support to continue clinical development and testing of efficacy of PvDBPII (50 μg dose) formulated with of GLA-SE (5 μg of GLA). The efficacy of PvDBPII/GLA-SE should now be tested in the *P. vivax* blood-stage challenge model^23^ in which the blood-stage growth of *P. vivax* in vivo can be quantitatively compared between a group that receives the vaccine and control group administered a control vaccine, such as the hepatitis B vaccine. In addition, the ability of PvDBPII/GLA-SE to protect against natural *P. vivax* infection may be tested in a Phase IIb field trial conducted in a *P. vivax* endemic region. These studies could provide proof-of-concept for a vaccine based on the PvDBP and validate the approach of targeting key receptor–ligand interactions that mediate host cell invasion to develop blood-stage vaccines for malaria.

## Materials and methods

### Vaccines used

PvDBPII was the investigational vaccine antigen used, while GeneVac-B (recombinant hepatitis B vaccine), provided by the Serum Institute of India, Pune, was used as a control vaccine. The design, production and preclinical testing of recombinant PvDBPII vaccine has been reported previously.^12^ Briefly, a codon-optimized synthetic PvDBPII gene encoding ~ 39 kDa protein was cloned into the pET28a (+) vector and the resultant plasmid used to transform the *E. coli* strain BLR(DE3) pLysS. PvDBPII was expressed in *E. coli* using a fed-batch fermentation strategy; cells were lysed and inclusion bodies were purified and solubilized. The recombinant PvDBPII was refolded using a rapid-dilution method and purified by cation-exchange chromatography followed by Mustang Q filtration. The final PvDBPII product was filter sterilized and stored at − 70 °C.

Clinical-grade recombinant PvDBPII was manufactured at Syngene International, India, and the drug product was vialed and released by Zydus Cadila, Ahmedabad, India, using cGMPs (current Good Manufacturing Practices). Each vial contained 0.5 mL of PvDBPII at a concentration of 200 µg/mL. The vials were stored < − 70 °C. Lot no. MASPN103 was used for the current study. This lot was released by the Central Research Institute, Kasauli, India, for use in the clinical trial. The adjuvant GLA-SE was manufactured at Gennova Pharmaceuticals, Pune. GLA-SE is a stable oil-in-water emulsion (SE) containing the immunological adjuvant GLA. Each vial contained 0.4 mL of GLA at a concentration of 20 µg/mL in 4% oil and these vials were stored at + 2 to + 8 °C.

Recombinant PvDBPII protein was formulated with the GLA-SE adjuvant in the pharmacy at the clinical trial site and administered to study subjects within 1 h of formulation preparation. PvDBPII was diluted with phosphate buffer saline to prepare 10 and 25 µg doses. For each dose, a volume of 0.5 mL of the formulated test vaccine was withdrawn for intramuscular administration to the subjects.

Single-dose 1 ml vials of GeneVac-B (hepatitis B vaccine), a ready-to-use suspension from the Serum Institute of India, Pune, was used as a control vaccine. The vaccine contains purified surface antigen (HBsAg) of the hepatitis B virus obtained by culturing genetically engineered *Hansenula polymorpha* yeast cells. The vaccine contains aluminium hydroxide as the adjuvant and thiomersal as a preservative.

### Study design, approvals and participants

The clinical trial was a single centre, phase I, randomized (3:1), controlled, dose-escalating, single-blind study for the assessment of safety and immunogenicity of the malaria vaccine candidate, PvDBPII/GLA-SE with Protocol No. MVDP/vivax/1/15/02/01 (CTRI/2016/09/007289). The study was conducted in the “Human Pharmacology Unit” of Syngene International Limited, Bengaluru, India. The trial was initiated only after receiving necessary written regulatory approvals from the Indian Drug Licensing Authority (CDSCO, India) and the Ethics Committee of the Sri Venkateshwara Hospital, Bengaluru. The study was carried out in accordance with the ethical principles laid down in the Declaration of Helsinki (Document adopted by the 59th WMA General Assembly, 2013), ICH guidelines for Good Clinical Practice, and in compliance with the approved study protocol.

The subjects were healthy, malaria-naive, male, Indian adults aged 18–45 years recruited from the volunteer database of healthy subjects available with the study site, Syngene International Limited, Bangalore. Subject recruitment was as per inclusion/exclusion criteria as detailed in the Supplementary Clinical Trial Protocol. All study procedures including screening were conducted after obtaining written Informed Consent from all subjects. The study involved administration of the investigational vaccine candidate, PvDBPII/GLA-SE, in three dose-escalating cohorts corresponding to three dosages of 10, 25 and 50 µg of PvDBPII with a constant dose of adjuvant GLA-SE (5 µg of GLA) as detailed in the Supplementary Clinical Trial Protocol. The vaccine was administered on days 0, 28 and 56 with follow-up till day 180. Recombinant hepatitis B vaccine (GeneVac-B provided by Serum Institute of India, Pune) was used as a control. In each cohort, 12 healthy subjects were enrolled. Following a 3:1 randomization scheme (SAS generated randomization schedule dated 21 April 2016, version 2.0), nine subjects received PvDBPII/GLA-SE, while three subjects received Hepatitis B vaccine in each cohort.

The randomization list was generated by the Biostatistician at Syngene using a reproducible, computer-generated block randomization schedule which was cohort specific to ensure allocation ratio 3:1 between investigational and control vaccine. Randomization list provided sequential codes linking vaccine assignment to subject number. A printed copy along with a soft copy of the randomization list was maintained by the biostatistician, human pharmacology unit and pharmacy at Syngene.

This clinical trial was designed as a single-blind study and subjects were blinded to the study treatment while the investigators, site staff, and data analysts were aware of the identity of the vaccine. While performing the immunogenicity assessments the scientists involved with ELISA and BIA were blinded.

### Study objectives

The primary objectives of the study were to assess the safety and reactogenicity of three different doses of malaria vaccine candidate, PvDBPII, formulated with GLA-SE in malaria naïve healthy male adults. The secondary objectives were to assess the immunogenicity of PvDBPII/GLA-SE by evaluating the humoral immune response against PvDBPII by ELISA, BIA and IFA assays.

### Safety analysis

Data on AEs were collected throughout the study at regular intervals. The subjects were closely monitored for immediate reactogenicity on the day of each dosing. Other safety data assessed included specified solicited symptoms collected by diary card during Days 0 7; unsolicited AEs collected on Days 0–28 after each dose and serious AEs collected Days 0–180. Laboratory assessments were done on Day 7 post each dose and then at the end-of-study visit by performing predefined laboratory parameter testing as listed in the Supplementary Clinical Trial Protocol. Solicited events, unsolicited events and changes in laboratory parameters are graded as per the standard US FDA Toxicity Grading Scale for Preventive Vaccine Clinical Trials.^24^ An independent data safety monitoring committee reviewed 7 days of safety data for each cohort escalation.

### Determination of anti-PvDBPII IgG antibody titres by ELISA assay

Immunogenicity analyses were conducted on the PP Population. Sera collected on Days 0, 28, 56, 84 and 180 were assessed for recognition of PvDBPII by ELISA assay. The humoral immune response to PvDBPII was assessed in reference to Day 0 samples collected before the first dose. In brief, Nunc-ImmunoMaxisorp™ 96-well plates were coated overnight at 4 °C with recombinant PvDBPII (2 µg/ml) in 100 μl of carbonate-bicarbonate coating buffer per well (Carbonate-Bicarbonate Buffer capsule, Sigma). The plates were washed three times using wash buffer and blocked with Starting Block T20 solution (Thermo) for 2 h at 37 °C. Antigen-coated wells were incubated in duplicate with test and reference standard sera diluted serially and incubated for 1 h at 37 °C. Plates were washed and bound antibodies were detected with horseradish peroxidase conjugate anti-human IgG secondary antibody (Sigma). The IgG subclasses IgG1, IgG2, IgG3 and IgG4 in serum samples were detected by biotin conjugated anti-human IgG1, IgG2, IgG3 and IgG4 (Sigma), respectively, followed by Avidin-HRP conjugate (Sigma). The assays were developed with TMB substrate (3,3′,5,5′-tetramethylbenzidine (Sigma)). The reaction was stopped with 2 N H_2_SO_4_ and the plates were read at 450 nm using a SpectramaxPlus384 microplate reader (Molecular Devices). Data were collected using Softmax Pro GXP v5. Naive sera from individuals not previously infected with malaria were used as negative control for the assays. Serum samples collected during screening that were reactive to PvDBPII by ELISA assay were pooled and used as reference positive control sera to generate the reference standard curve. The reference positive control sera pool was assigned 940 ELISA U/mL, which was approximately equivalent to the reciprocal of the dilution that yields an optical density of ~ 1 at a wavelength of 450 nm (OD_450_) by ELISA assay. The standard curve was then used to convert absorbance values of individual test sera (diluted to yield absorbance within the linear range of the standard curve) to U/mL using ADAMSEL FLP b040 software (Ed Remarque© 2009, BPRC, The Netherlands). Test samples that did not fall within an acceptable OD_405_ range were retested at an alternate dilution. A response for different antibody subclasses in a serum sample was considered positive if an OD value above the cut off (arithmetic mean of negative controls plus 3 standard deviations) was achieved.

### Anti-PvDBPII IgG antibodies by IFA assay

IFA assay was used to test recognition of native PvDBPII antigen in *P. vivax* schizonts by subject serum samples. Anti-PvDBPII mouse sera (1:50) and reference positive control human sera (1:50) were used as positive controls. Naïve human sera (1:50) and pre-immune mouse sera (1:50) were used as negative controls. Test sera from 3 time points, Day 0 (V1), Day 84 (V16) and Day 180 (V17) were tested at dilution of 1:50. All primary and secondary antibody incubations were carried out for 30 min at room temperature. Fluorescently labelled (fluorescein isothiocyanate, FITC)-goat anti-human IgG or FITC-conjugated goat anti-mouse IgG secondary antibodies were used to detect human and mouse serum antibodies, respectively. After staining, slides were mounted with anti-Fade plus DAPI (4’-6-Diamidno-2-phenylindole, to counterstain parasite nuclei) and were viewed under oil immersion using a confocal scanning laser microscope (× 100).

### Expression and purification of DARC‐Fc

A plasmid encoding DARC‐Fc was generated by ligating the first 60 codons of human DARC (FyB) to sequences encoding Fc region of human IgG (DARC‐Fc) in a mammalian expression vector.^25,26^ Recombinant DARC-Fc was produced by co‐transfecting 293 T cells with plasmids encoding DARC‐Fc fusion protein and human sulfotransferase. Recombinant DARC-Fc protein was purified from cell culture supernatants by affinity chromatography using Protein A. The concentration of purified DARC‐Fc was determined by bicinchoninic acid.

### ELISA-based binding-inhibition assay

The ability of anti-PvDBPII sera to inhibit PvDBPII-DARC interaction was measured using an ELISA-based BIA assay as described previously.^10^ Recombinant DARC-Fc (1 µg/well) was coated on to a 96-well plate and the plate was blocked using blocking buffer T20 (Thermo). Recombinant PvDBPII in a range of 0.8–25 ng/ml was used to generate a PvDBPII standard curve using a four-parameter logistic curve. CT serum samples were analysed at different dilutions (1:10 to 1:5000) and each of the diluted sera was then incubated with 25 ng/ml PvDBPII test antigen for 1 h at room temperature. In addition, combinations of PvDBPII test antigen (fixed conc. of 25 ng/ml) and fixed dilution (1:10) of positive control human sera was used as positive control and naïve human reference sera (1:10) was used as negative control. The reaction mixture and standard dilution of PvDBPII antigen were then added to the wells of DARC-coated plate. PvDBPII antigen bound to DARC was probed with anti-PvDBPII polyclonal rabbit sera and detected with anti-Rabbit IgG HRP-conjugated secondary antibody. The assay was developed using the chromogenic substrate TMB (3,3′,5,5′-tetramethylbenzidine) and colour development was stopped with sulfuric acid (H_2_SO_4_). Absorbance was measured at a wavelength of 450 nm, with background subtraction at 630 nm. The amount of bound PvDBPII was estimated by converting OD values to protein concentrations using the PvDPBII standard/calibration curve. The obtained protein concentration values were used to calculate % binding and % binding inhibition of PvDBPII-DARC interaction. Percent inhibition at each dilution was determined as (100 %–% binding). A curve of % inhibition versus serum dilution was used to determine 50% binding-inhibition titre for each serum sample.

### Statistical analysis

This is a proof of safety and immunogenicity study therefore no formal sample size calculations were performed. No statistical hypothesis was formulated for this study and only descriptive statistics was performed for all parameters. Comparisons between groups were performed with Fisher’s exact tests. To delineate differences in antibody responses between the three dose groups, a Kruskal–Wallis test was performed for ELISA results. The correlation between antibody level and binding-inhibitory activity was tested using a Spearman’s rank test. Vaccine response distributions are represented by reverse cumulative distributions.^27^ All statistical tests were two-sided and differences with a *p*-value < 0.05 were considered significant. Statistical Analysis System software (SAS version 92) was used for these analyses. Figures are plotted using GraphPad Prism version 6.07 (GraphPad Software, Inc., California, USA).

### URL in registry database

http://ctri.nic.in/Clinicaltrials/pdf_generate.php?trialid=15654&EncHid=&modid=&compid=%27,%2715654det%27

## Electronic supplementary material

Supplementary information

## Data Availability

Clinical data that support the findings of this study are available through the corresponding author upon reasonable request.

## Electronic supplementary material

**Supplementary information** accompanies the paper on the *npj Vaccines* website (10.1038/s41541-018-0083-3).
