# Supplementary material for: Malaria vaccine candidate based on Duffy-binding protein elicits strain transcending functional antibodies in a Phase I trial
Source: NPJ Vaccines. 2018 Sep 28;3:48. doi: 10.1038/s41541-018-0083-3 (PMC6162314; doi:10.1038/s41541-018-0083-3)
Supplement: Supplementary file 1 — Supplementary information [file 41541_2018_83_MOESM1_ESM.docx]

**Supplementary Information**

**Supplementary Table S1.** **Incidence local and systemic solicited adverse events in groups receiving 10µg, 25µg or 50µg PvDBPII vaccine and 20µg Hepatitis B vaccine**

| Description | **PvDBPII**  **(10µg) N=9 n(%)** | **PvDBPII**  **(25 µg) N=9 n(%)** | **PvDBPII**  **(50 µg) N=9 n(%)** | **Hepatitis B**  **(20 µg) N=9 n(%)** |
| --- | --- | --- | --- | --- |
| Solicited Local Adverse Events | | | | |
| Pain | 1 (11.11 )* | 0.00 (0) | 0.00 (0) | 0.00 (0) |
| Swelling | 0.00 (0) | 0.00 (0) | 0.00 (0) | 0.00 (0) |
| Erythema | 0.00 (0) | 0.00 (0) | 0.00 (0) | 0.00 (0) |
| Induration | 0.00 (0) | 0.00 (0) | 0.00 (0) | 0.00 (0) |
| Tenderness | 0.00 (0) | 0.00 (0) | 0.00 (0) | 0.00 (0) |
| Solicited Systemic Adverse Events | | | | |
| Fever | 0.00 (0) | 0.00 (0) | 0.00 (0) | 0.00 (0) |
| Vomiting | 0.00 (0) | 0.00 (0) | 0.00 (0) | 0.00 (0) |
| Diarrhoea | 0.00 (0) | 0.00 (0) | 0.00 (0) | 0.00 (0) |
| Headache | 0.00 (0) | 0.00 (0) | 0.00 (0) | 0.00 (0) |
| Fatigue | 0.00 (0) | 0.00 (0) | 0.00 (0) | 0.00 (0) |
| Myalgia | 0.00 (0) | 0.00 (0) | 0.00 (0) | 0.00 (0) |
| Note: N= Total number of subjects randomized, n= number of subjects assigned for individual analysis; %=(n/N)100;Exact Binomial proportion and 95% CIs are calculated using Clopper-Pearson Method used in SAS  * Mild in severity | | | | |

**Supplementary Table S2. Summary of unsolicited adverse events(s) by SOC and Preferred term for all study groups-ITT Population**

| **System Organ Class** | **Preferred Term** | **PvDBPII (10µg) N=9 n(%)** | **PvDBPII (25µg) N=9 n(%)** | **PvDBPII (50µg) N=9 n(%)** | **Hepatitis B (20 µg) N=9 n(%)** |
| --- | --- | --- | --- | --- | --- |
| Total Adverse Events reported |  | 13 | 15 | 19 | 20 |
| Investigations | Alanine aminotransferase increased | 0 (0.00) | 2 (22.22) | 1 (11.11) | 1 (11.11) |
|  | Aspartate aminotransferase increased | 1 (11.11) | 2 (22.22) | 0 (0.00) | 1 (11.11) |
|  | Blood bilirubin increased | 0 (0.00) | 0 (0.00) | 1 (11.11) | 2 (22.22) |
|  | Blood glucose decreased | 0 (0.00) | 0 (0.00) | 1 (11.11) | 1 (11.11) |
|  | Blood glucose increased | 2 (22.22) | 3 (33.33) | 4 (44.44) | 5 (55.56) |
|  | Haemoglobin decreased | 5 (55.56) | 4 (44.44) | 2 (22.22) | 2 (22.22) |
| Metabolism and nutrition disorders | Hyperkalaemia | 2 (22.22) | 2 (22.22) | 2 (22.22) | 1 (11.11) |
|  | Hypernatremia | 1 (11.11) | 2 (22.22) | 5 (55.56) | 3 (33.33) |
| Skin and subcutaneous tissue disorders | Rash | 1 (11.11) | 0 (0.00) | 0 (0.00) | 0 (0.00) |
| System Organ Class (SOC) and Preferred Term (PT) coded as per MedDRA Version 18.1. Note:  If a subject experiencing the same AEs multiple times will be counted only once for the corresponding PT N= Total number of subjects randomized, n= number of subjects assigned for individual analysis. %=(n/N)100 | | | | | |

**Supplementary Table S3**. CONSORT 2010 checklist of information to include when reporting a randomised trial.

| **Section/Topic** | **Item no.** | **Standard CONSORT Checklist item** | **Page no.** |
| --- | --- | --- | --- |
| **Title and abstract** | | |  |
|  | 1a | Identification as a randomised trial in the title | 1 |
|  | 1b | Structured summary of trial design, methods, results, and conclusions (for specific guidance see CONSORT for abstracts[3]) | 2 |
| **Introduction** | | |  |
| Background and objectives | 2a | Scientific background and explanation of rationale | 3-4 |
|  | 2b | Specific objectives or hypotheses | 4 |
| **Methods** | | |  |
| Trial design | 3a | Description of trial design (such as parallel, factorial) including allocation ratio | 19, 20 |
|  | 3b | Important changes to methods after trial commencement (such as eligibility criteria), with reasons | NA |
| Participants | 4a | Eligibility criteria for participants | 19 |
|  | 4b | Settings and locations where the data were collected | 19 |
| Interventions | 5 | The interventions for each group with sufficient details to allow replication, including how and when they were actually administered | 19, 20 |
| Outcomes | 6a | Completely defined pre-specified primary and secondary outcome measures, including how and when they were assessed | 20 -24 |
|  | 6b | Any changes to trial outcomes after the trial commenced, with reasons | NA |
| Sample size | 7a | How sample size was determined | 24 |
|  | 7b | When applicable, explanation of any interim analyses and stopping guidelines | No interim analysis was done |
| Randomisation: | | |  |
| Sequence generation | 8a | Method used to generate the random allocation sequence | 20 |
|  | 8b | Type of randomisation; details of any restriction (such as blocking and block size) | 20 |
| Allocation concealment mechanism | 9 | Mechanism used to implement the random allocation sequence (such as sequentially numbered containers), describing any steps taken to conceal the sequence until interventions were assigned | NA |
| Implement-ation | 10 | Who generated the random allocation sequence, who enrolled participants, and who assigned participants to interventions |  |
| Blinding (masking) | 11a | If done, who was blinded after assignment to interventions (for example, participants, care providers, those assessing outcomes) and how | 19, 20 |
|  | 11b | If relevant, description of the similarity of interventions | NA |
| Statistical methods | 12a | Statistical methods used to compare groups for primary and secondary outcomes | 24, 25 |
|  | 12b | Methods for additional analyses, such as subgroup analyses and adjusted analyses | NA |
| **Results** | | |  |
| Participant flow  (a diagram is strongly recommended) | 13a | For each group, the numbers of participants who were randomly assigned, received intended treatment, and were analysed for the primary outcome | 6 and Figure 1 |
|  | 13b | For each group, losses and exclusions after randomisation, together with reasons | 6 |
| Recruitment | 14a | Dates defining the periods of recruitment and follow-up | 6 |
|  | 14b | Why the trial ended or was stopped | 6 |
| Baseline data | 15 | A table showing baseline demographic and clinical characteristics for each group | Table 1 |
| Numbers analysed | 16 | For each group, number of participants (denominator) included in each analysis and whether the analysis was by original assigned groups | 6, Tables 1 and 2 on pages 33, 34 |
| Outcomes and estimation | 17a | For each primary and secondary outcome, results for each group, and the estimated effect size and its precision (such as 95% confidence interval) | 6- 9, 10 and 12 |
|  | 17b | For binary outcomes, presentation of both absolute and relative effect sizes is recommended | NA |
| Ancillary analyses | 18 | Results of any other analyses performed, including subgroup analyses and adjusted analyses, distinguishing pre-specified from exploratory | NA |
| Harms | 19 | All important harms or unintended effects in each group (for specific guidance see CONSORT for harms) | NA |
| **Discussion** | | |  |
| Limitations | 20 | Trial limitations, addressing sources of potential bias, imprecision, and, if relevant, multiplicity of analyses | NA |
| Generalisability | 21 | Generalisability (external validity, applicability) of the trial findings | NA |
| Interpretation | 22 | Interpretation consistent with results, balancing benefits and harms, and considering other relevant evidence | 14, 15, 16, 17 |
| **Other information** | | |  |
| Registration | 23 | Registration number and name of trial registry | 1 |
| Protocol | 24 | Where the full trial protocol can be accessed, if available | Protocol submitted with the manuscript |
| Funding | 25 | Sources of funding and other support (such as supply of drugs), role of funders | 26, 28 |

**
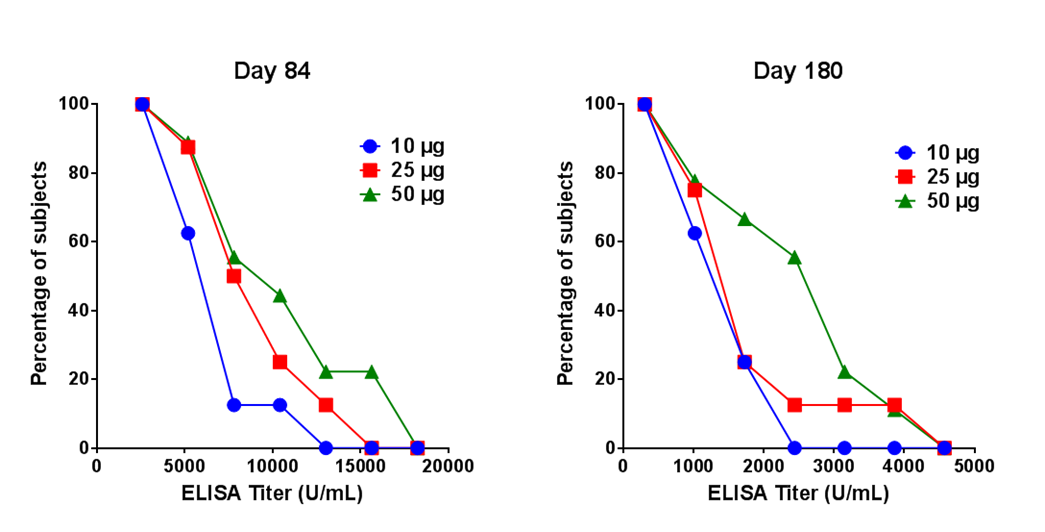
**

**Supplementary Figure S1.** Reverse cumulative distribution curve for PvDBPII antibody titer measured by ELISA at Day 84 and 180 for all PvDBPII dose groups.


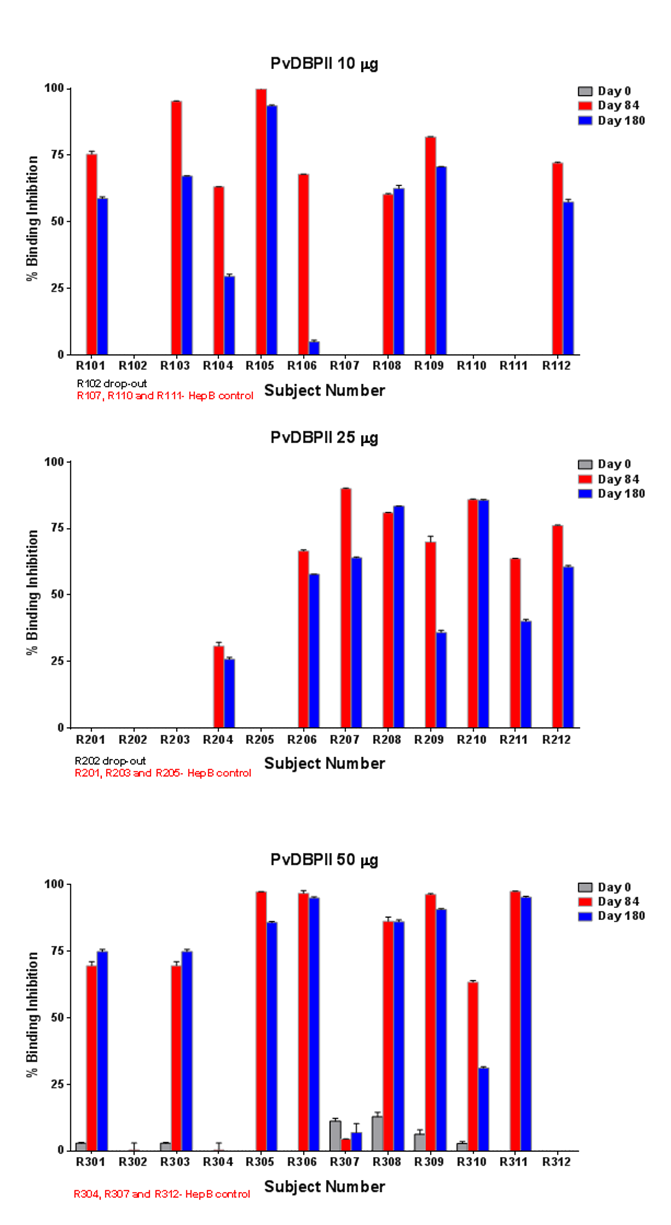


**Supplementary Figure S2.** Ability of PvDBPII specific antisera to inhibit binding of recombinant PvDBPII (Sal I) to DARC using an ELISA-based binding inhibition assay. Mean percentage binding inhibition at Day0, Day 84 and Day 180 for PvDBPII 10, 25 and 50 µg dose groups tested at 1:10 sera dilution (PP Population).

**
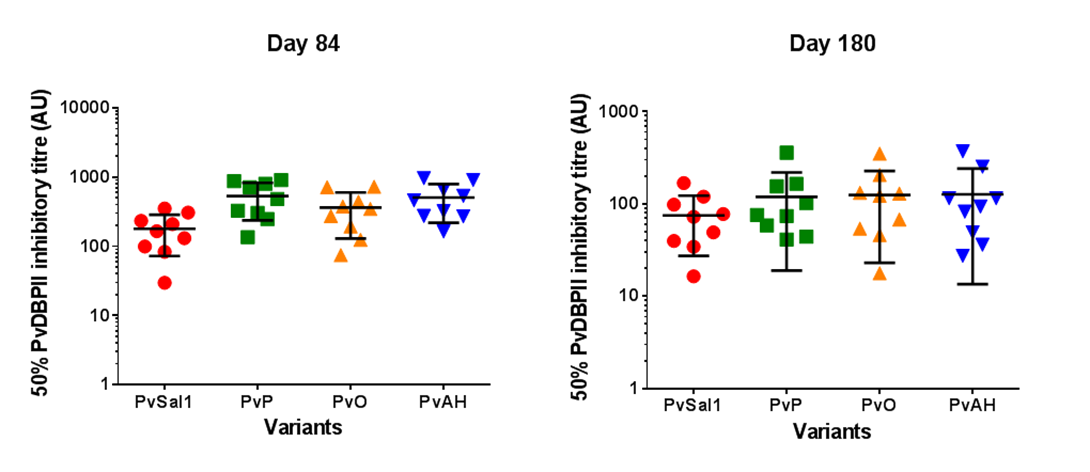
Supplementary Figure S3**. Anti-PvDBPII-specific binding inhibitory Ab titer to different variants of PvDBPII (Sal I, O, P and AH).Variant-specific binding inhibitory activities at Day 84 and Day 180 were tested at sera dilutions from 1:10 to 1:1,000 to determine 50% binding inhibition titres.


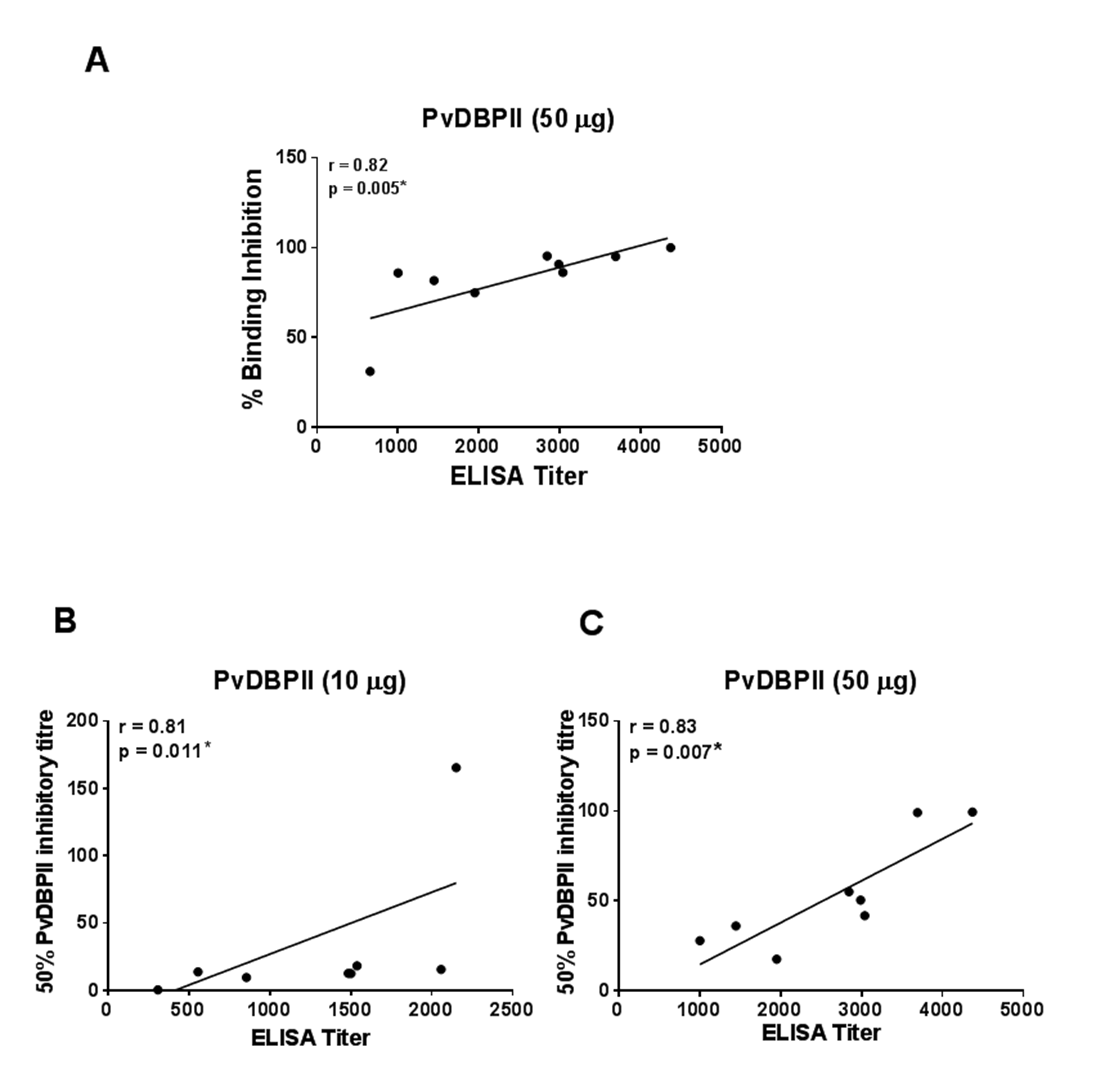


**Supplementary Figure S4.** Correlation between ELISA and Binding Inhibtion (PP Population). A. Correlation between ELISA titer and Percent Binding Inhibition (1:10) at Day 180 for PvDBPII 50 µg dose group. B and C, Correlation between ELISA titer and 50% PvDBPII Binding Inhibitory titer at Day 180 for PvDBPII10 and 50 µg dose groups.
